# Efficacy of Acupuncture in Managing Radiation-Induced Xerostomia: An Updated Meta-Analysis

**DOI:** 10.7150/ijms.110366

**Published:** 2025-05-31

**Authors:** Ru-Yin Tsai, Sheng-Yi Lin, Chin-Chang Chen, Yao Hsiao

**Affiliations:** 1 Department of Anatomy, School of Medicine, Chung Shan Medical University, Taichung 40201, Taiwan. geoge6211@csmu.edu.tw (CCC); shengyi@csmu.edu.tw (SYL).; 2 Department of Medical Education, Chung Shan Medical University Hospital, Taichung 40201, Taiwan.; 3 School of Medicine, Chung Shan Medical University, Taichung 40201, Taiwan. s1001085@gm.csmu.edu.tw.

**Keywords:** cancer, systematic reviews, salivary flow, dry mouth, quality of life.

## Abstract

**Background:** Xerostomia, or dry mouth, frequently affects head and neck cancer patients receiving radiotherapy, leading to discomfort and impacting daily functions such as speaking and swallowing. Conventional treatments may offer limited relief and are often accompanied by undesirable side effects. Acupuncture, as a non-pharmacological intervention, is increasingly explored for its potential to mitigate xerostomia symptoms.

**Objective:** This systematic review and meta-analysis aim to assess the effectiveness of acupuncture in improving symptoms and quality of life in patients experiencing radiation-induced xerostomia.

**Methods:** A thorough literature search was conducted across several databases, including MEDLINE, Embase, Cochrane Central, and Web of Science, up to the current year. We included randomized controlled trials (RCTs) that evaluated acupuncture's impact on salivary flow and symptom relief in adults with radiation-induced xerostomia. Primary outcomes were changes in salivary flow, with secondary outcomes including patient-reported symptom severity and quality of life metrics. The risk of bias was evaluated, and data were synthesized using a random-effects model.

**Results:** A total of 11 RCTs involving 1271 participants were included in the analysis. The pooled data showed a moderate increase in salivary flow in the acupuncture group, effective in both resting and stimulated conditions. Additionally, acupuncture demonstrated significant benefits in reducing xerostomia symptoms and improving quality of life scores compared to control interventions. Subgroup analysis revealed that traditional acupuncture was more effective than Transcutaneous Electrical Nerve Stimulation (TENS).

**Conclusions:** The findings suggest that acupuncture may be an effective complementary treatment for radiation-induced xerostomia, offering relief from dry mouth symptoms and potentially improving quality of life. Further research should focus on standardizing acupuncture protocols to confirm these benefits across diverse patient populations.

## Introduction

Xerostomia, commonly referred to as dry mouth, is a frequently reported complication among patients undergoing radiotherapy for head and neck cancers 1. It is essential to distinguish between xerostomia and hyposalivation: while xerostomia describes the subjective sensation of oral dryness, hyposalivation refers to the objectively measured reduction in salivary flow 2. Although the two often coexist, patients may report xerostomia despite normal salivary output, and vice versa, highlighting the need to address both subjective symptoms and physiological markers in clinical management 3. Radiation-induced xerostomia results primarily from damage to the salivary glands, leading to diminished salivary production 4. This condition can severely impair oral functions such as speaking, chewing, and swallowing, thereby diminishing quality of life and nutritional status 5. Furthermore, insufficient saliva compromises the oral microbiome, increasing susceptibility to dental caries, mucosal infections, and overall oral discomfort, complicating recovery and long-term survivorship 4.

While pharmacological agents, such as pilocarpine, are available for managing xerostomia, their effectiveness is often limited and may come with side effects like sweating and gastrointestinal discomfort 6. As a result, there is growing interest in exploring non-pharmacological therapies like acupuncture, which has shown potential in alleviating xerostomia symptoms by enhancing residual salivary gland function through neurovascular stimulation 3, 5. Acupuncture is thought to influence salivary secretion pathways and may stimulate the autonomic nervous system to increase salivary flow, thereby potentially offering symptom relief without adverse effects 7, 8.

Several studies and preliminary reviews suggest that acupuncture may improve salivary flow and reduce xerostomia severity in patients' post-radiotherapy 9, 10. However, these reviews have often been limited by small sample sizes and variation in study methodologies. To provide a clearer understanding of acupuncture's effectiveness in this context, a comprehensive and updated meta-analysis is warranted. This study aims to consolidate existing RCTs to evaluate the efficacy of acupuncture in managing radiation-induced xerostomia, offering a more definitive assessment of its role in supporting recovery and enhancing patient quality of life.

## Materials and Methods

### Data sources and selection criteria

Our study conducted a thorough search of RCTs evaluating the effects of acupuncture on radiation-induced xerostomia. We systematically searched multiple databases, including PubMed, Embase, the Cochrane Library, and Web of Science, for studies published up to September 2024. The search strategy incorporated terms such as "(acupuncture OR transcutaneous electrical nerve stimulation OR TENS OR pharmacopuncture) AND (radiation OR radiotherapy OR radiation therap* OR therap*, radiation OR radiation treatment*) AND (xerostomia OR hyposalivation OR Mouth Dryness)," focusing exclusively on clinical trials involving human participants. The review process strictly followed the Preferred Reporting Items for Systematic Reviews and Meta-Analyses (PRISMA) guidelines. All identified articles were screened, and their reference lists were examined for additional relevant studies. Exclusions included case reports, technical papers, conference abstracts, reviews, letters, editorials, and preclinical or laboratory-based studies. This meta-analysis has been registered with PROSPERO under registration number CRD42024581756.

### Selection of studies

**Figure 1:**
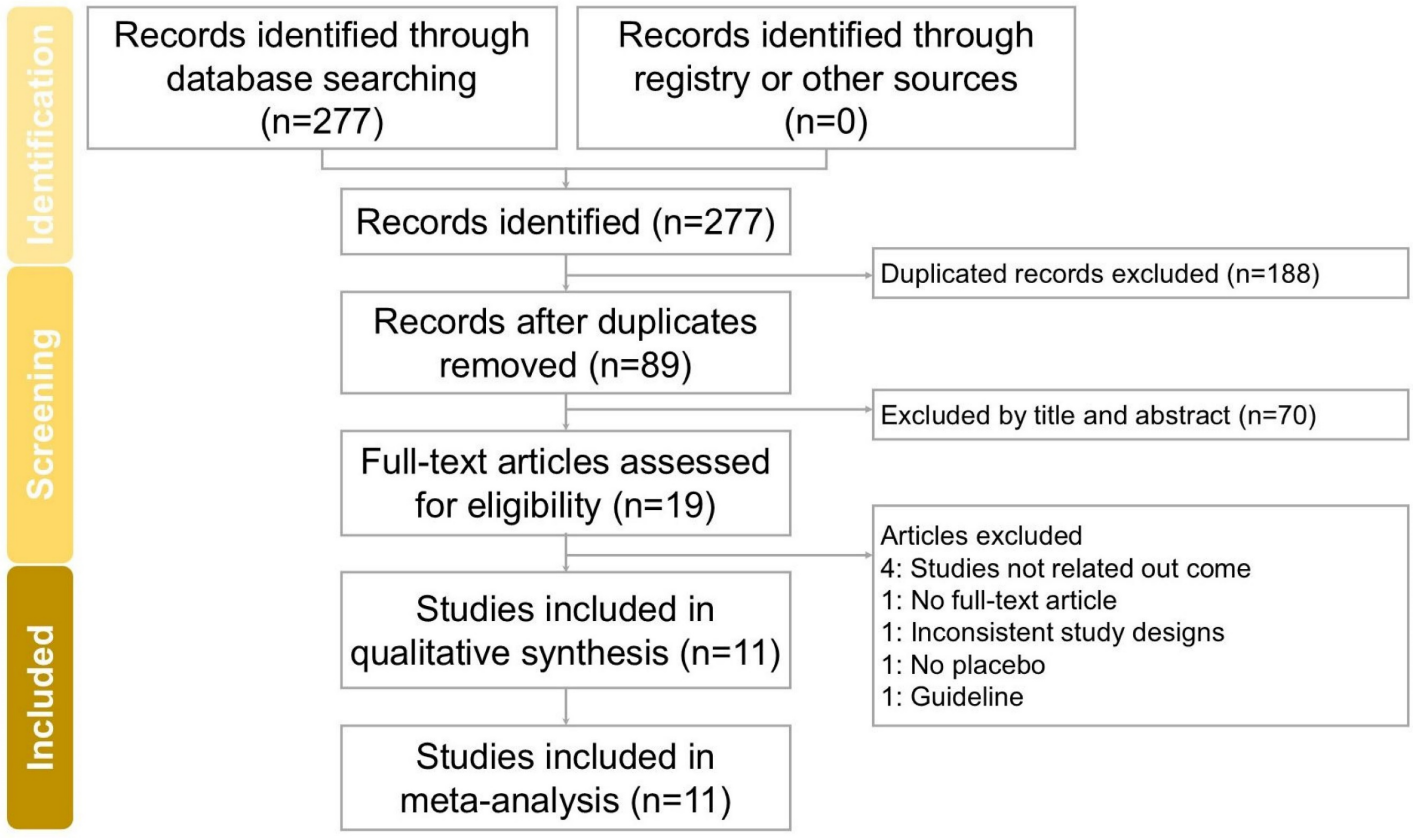
** Flowchart depicting the study selection process for this systematic review and meta-analysis on acupuncture interventions for radiation-induced xerostomia.** Of the 277 records initially identified, 11 studies fulfilled the inclusion criteria and were included in the final analysis.

The screening and assessment of studies were performed independently by two researchers, with a third researcher supervising the process to ensure accuracy and consistency. To support a comprehensive review, physical copies of all eligible articles were obtained and meticulously analyzed. The full study selection process is detailed in the PRISMA flow diagram (Figure 1).

### Data extraction

Data extraction was carried out independently by two authors using a standardized template developed in accordance with the Cochrane Handbook guidelines 11. The extracted information included key study details, such as author names, publication year, study location, participant eligibility criteria, demographic characteristics (e.g., total sample size and age range), study design, intervention specifics, reported outcomes, and the methods used for outcome assessment.

### Outcomes

The primary outcomes of this study focused on assessing resting salivary flow rates (RSF) and stimulated salivary flow rates (SSF). Secondary outcomes included xerostomia questionnaire scores (XQ) and measures of quality of life (QoL).

### Assessment of methodological quality

Two independent researchers conducted a thorough evaluation of potential biases in the included studies using the Cochrane Collaboration's Risk of Bias tool to assess methodological quality. Discrepancies in their assessments were resolved through detailed discussions with a third reviewer until a consensus was achieved. Studies were deemed to have a high risk of bias if significant concerns were identified in one or more domains outlined by the assessment tool.

### Data analysis

Quantitative analysis of the selected studies was performed using the Standardized Mean Difference (SMD) and 95% Confidence Intervals (CIs) to compare outcomes between intervention and control (placebo) groups. The SMD values were pooled using a random-effects model to account for inter-study variability. Statistical analyses were conducted with Comprehensive Meta-Analysis software, version 3 (Version 3.0 Biostat, Englewood, NJ, USA). Heterogeneity was evaluated using the *I²* statistic, with values exceeding 50% considered indicative of substantial heterogeneity. Publication bias was assessed through funnel plot visualization and Egger's regression test, with statistical significance defined as *p* < 0.05 for most analyses and *p* < 0.10 for publication bias assessment. Subgroup analyses were conducted to investigate potential sources of heterogeneity, while sensitivity analyses were performed by sequentially excluding individual studies to evaluate the stability of the overall results.

## Results

### Study search and characteristics of included patients

The trial screening and selection process is depicted in Figure 1. An initial search across four databases (PubMed, Embase, Cochrane Library, and Web of Science) identified 277 trials. After removing duplicates, 89 trials proceeded to title and abstract screening, resulting in the exclusion of 19 studies. A detailed full-text review of the remaining 19 trials led to the exclusion of four additional studies due to unavailable full texts (1 trial 12), irrelevant outcomes (4 trials 13-16), guideline (1 trial 17), no placebo (1 trial 18), and inconsistent study designs (1 trial 19). Ultimately, 11 trials 3, 5, 7-10, 20-24 met the inclusion criteria and were incorporated into this meta-analysis. All included studies were published in English. Table 1 summarizes the key characteristics of these trials, which were conducted between 1996 and 2024, involving a total of 1159 participants, with sample sizes ranging from 6 to 115 per study. Each trial assessed the effectiveness of acupuncture in managing radiation-induced xerostomia.

### Quality assessment

**Figure 2:**
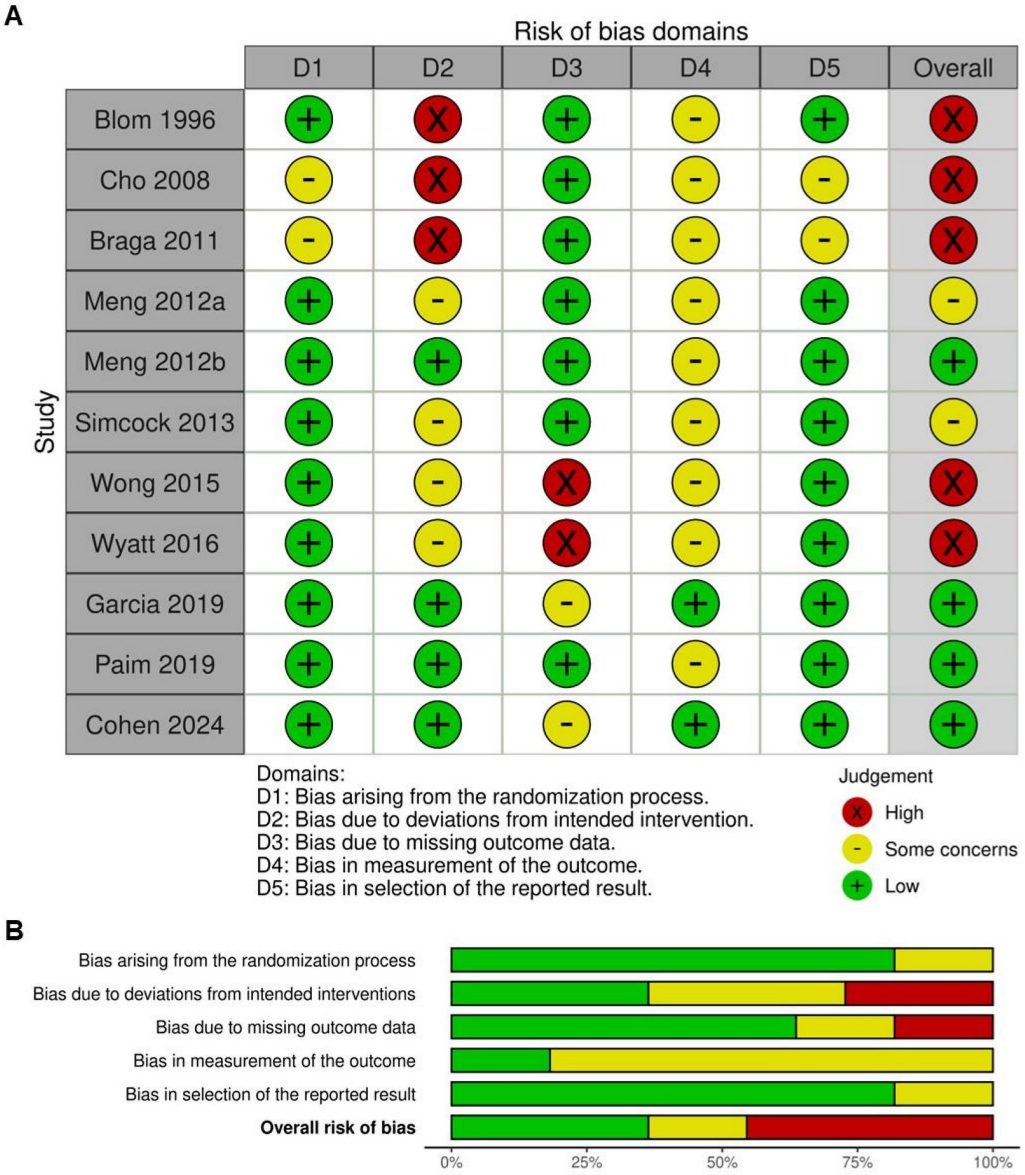
** Assessment of methodological quality of the included trials.** (A) Risk of bias for each included study. (B) The overall summary of bias of the ten studies.

The quality assessment of the included trials is presented in Figure 2A and 2B. Of the studies analyzed, four were determined to have a low risk of bias, attributed to their accuracy in reporting results 3, 5, 22, 24. Regarding randomization methods, nine trials were classified as having a low risk of bias 3, 5, 7, 8, 10, 20, 22-24. However, some concerns were identified in four trials 7, 8, 10, 23 and three trials 9, 20, 21, were judged to have a high risk of bias due to deviations from the intended interventions. Furthermore, all trials raised concerns over the absence of blinding, as outcome assessors were aware of treatment allocations, potentially introducing detection bias. Additionally, two trials 9, 21 presented some concerns regarding selective reporting, as they lacked a clearly pre-registered protocol or predefined analysis plan, increasing the likelihood of outcome reporting bias.

### Impact of acupuncture on RSF and SSF

**Figure 3:**
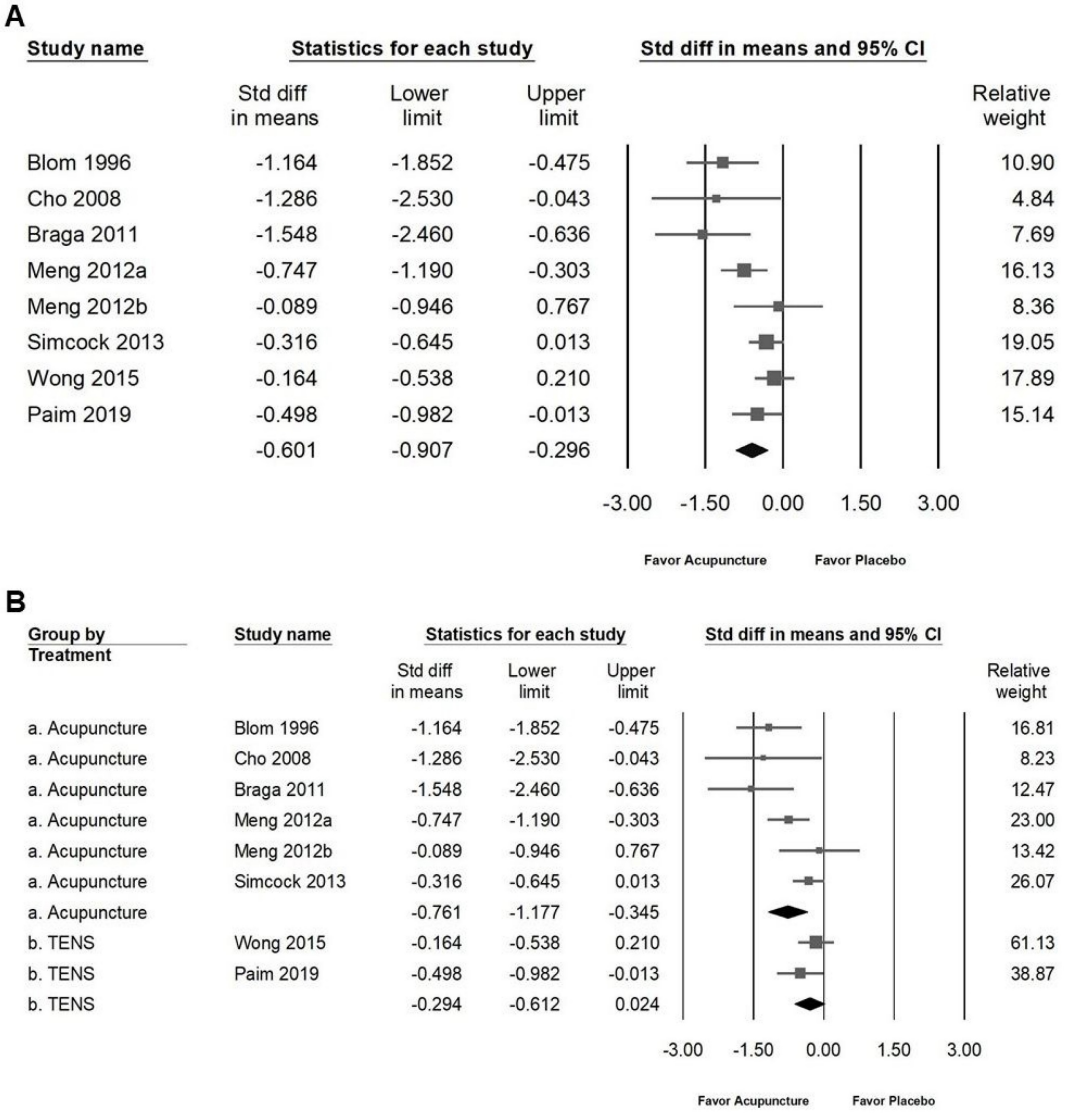
** Visualization of the effects of acupuncture on resting salivary flow.** Panel (A) highlights the overall impact on resting salivary flow, while panel (B) presents subgroup analyses by treatment type. In the forest plots, squares indicate standardized mean differences favoring acupuncture (shifted to the left), with horizontal lines representing the 95% confidence intervals. The diamond symbol at the bottom reflects the pooled effect size.

**Figure 4:**
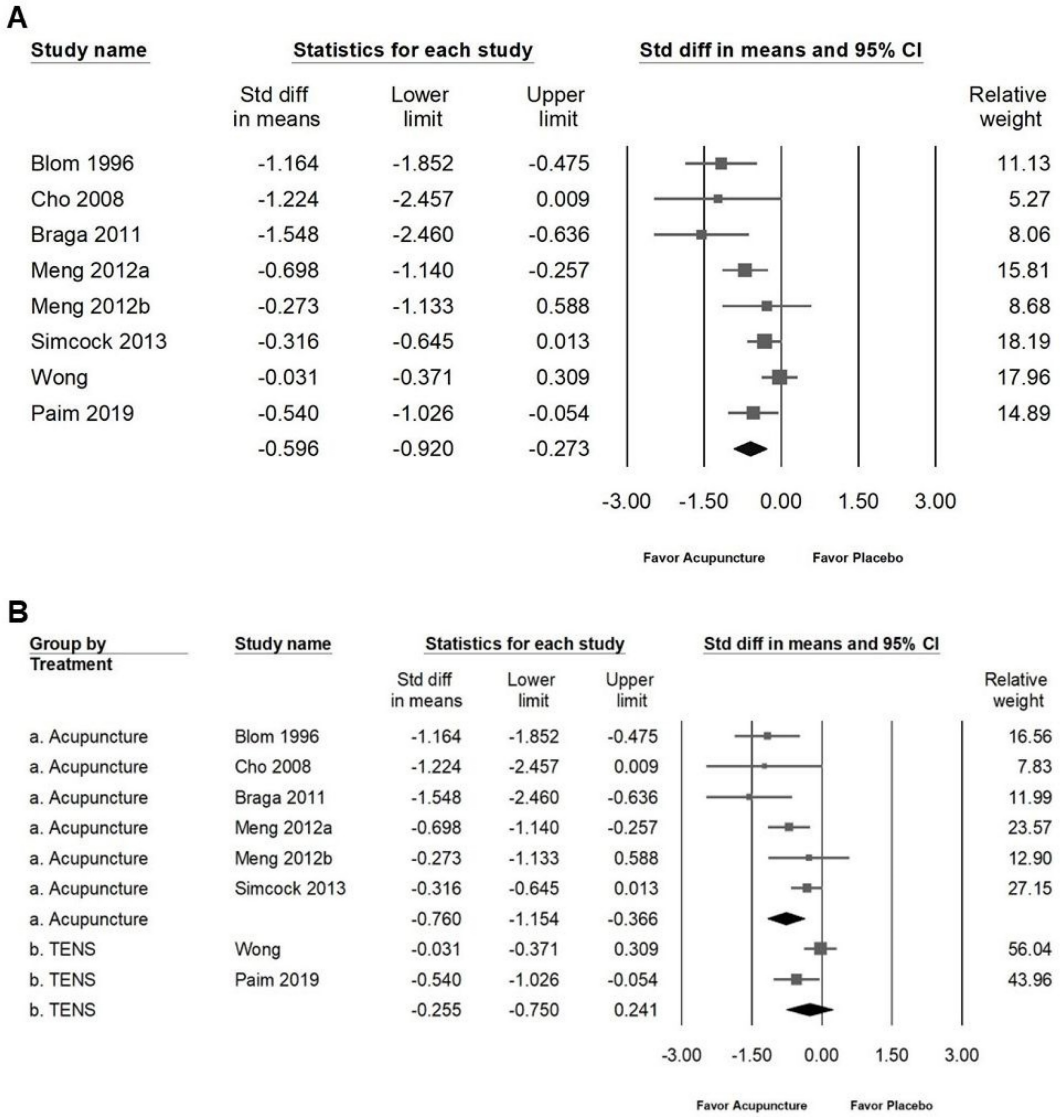
** Illustration of the effects of acupuncture on stimulated salivary flow.** Panel (A) shows the overall impact on stimulated salivary flow, while panel (B) provides subgroup analyses by treatment type. In the forest plots, squares represent standardized mean differences favoring acupuncture (shifted to the left), with horizontal lines indicating the 95% confidence intervals. The diamond symbol at the bottom summarizes the pooled effect size.

The intervention demonstrated a modest effect on improving salivary flow rates in patients with radiation-induced xerostomia. As illustrated in Figure 3A, the SMD for moderate improvement in resting salivary flow (RSF) was -0.601 (95% CI: -0.907 to -0.296; *I²* = 57.68%, *p* = 0.021). Subgroup analysis (Figure 3B) showed that acupuncture had a moderate effect on RSF (SMD: -0.761, 95% CI: -1.177 to -0.345; *I²* = 60.12%, *p* = 0.028), whereas no significant effect was observed for TENS (SMD: -0.294, 95% CI: -0.612 to 0.024; *I²* = 12.16%, *p* = 0.286). Similarly, the intervention showed moderate improvement in stimulated salivary flow (SSF), as indicated in Figure 4A, with an SMD of -0.596 (95% CI: -0.920 to -0.273; *I²* = 63.54%, *p* = 0.008). Subgroup analysis (Figure 4B) revealed that acupuncture had a significant moderate effect on SSF (SMD: -0.760, 95% CI: -1.154 to -0.366; *I²* = 55.81%, *p* = 0.045), while no significant effect was identified for TENS (SMD: -0.255, 95% CI: -0.750 to 0.241; *I²* = 64.73%, *p* = 0.092). These findings suggest that acupuncture is moderately effective in enhancing both RSF and SSF among patients with radiation-induced xerostomia, whereas TENS does not exhibit significant efficacy in this context.

### Effect of acupuncture on XQ and QoL

**Figure 5:**
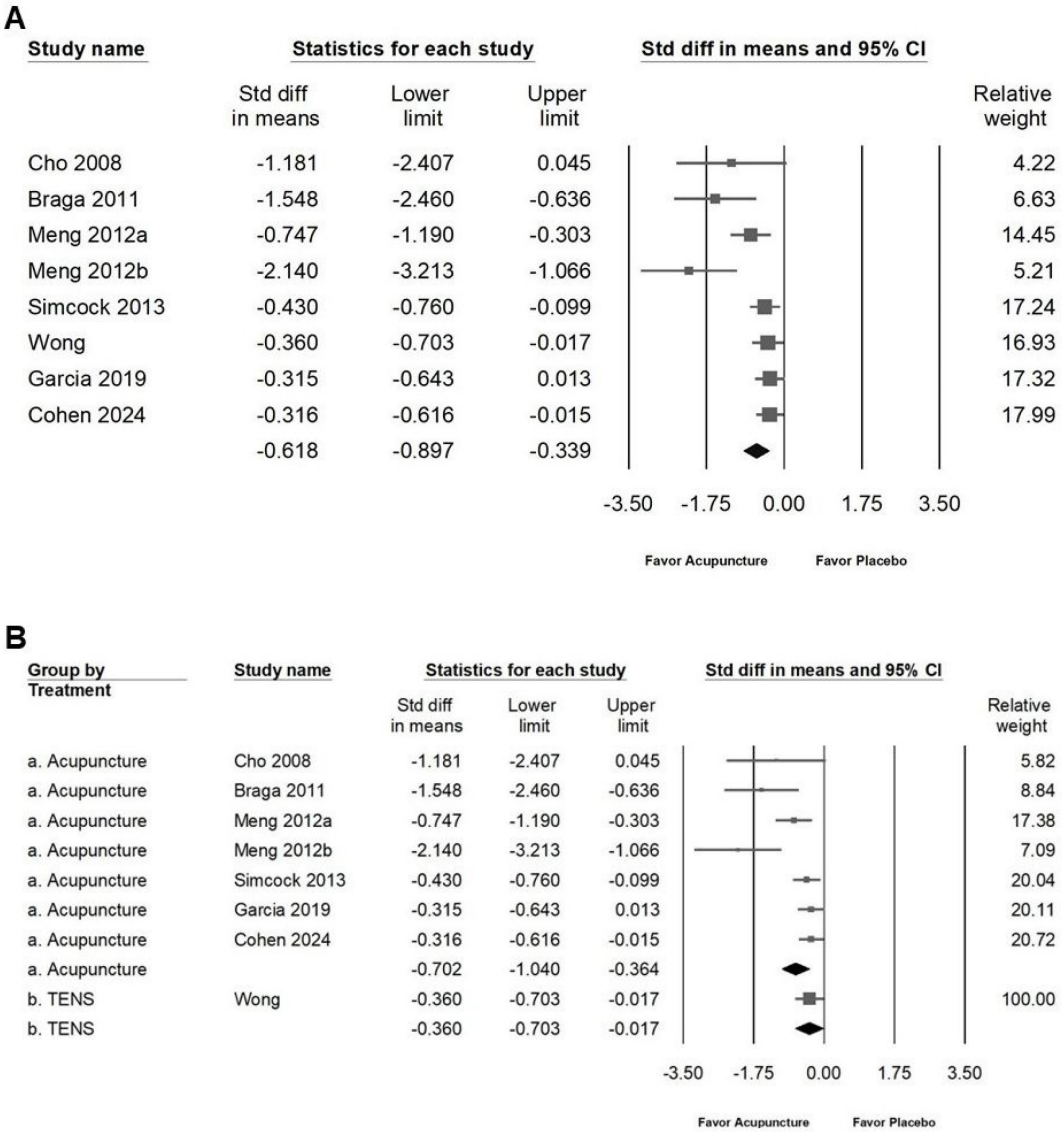
** Depict of the effects of acupuncture on xerostomia questionnaire scores.** Panel (A) presents the overall impact on xerostomia questionnaire scores, while panel (B) includes subgroup analyses by treatment type. In the forest plots, squares represent standardized mean differences favoring acupuncture (shifted to the left), with horizontal lines showing the 95% confidence intervals. The diamond symbol at the bottom reflects the pooled effect size.

**Figure 6:**
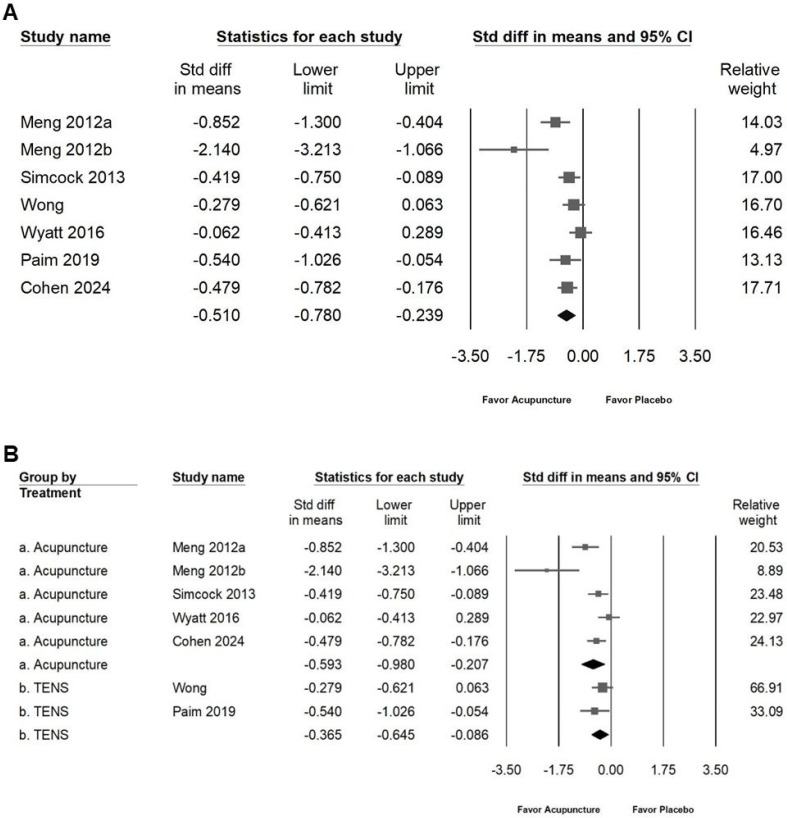
** Depiction of the effects of acupuncture on quality of life.** Panel (A) presents the overall impact on quality of life, while panel (B) includes subgroup analyses by treatment type. In the forest plots, squares indicate standardized mean differences favoring acupuncture (shifted to the left), with horizontal lines representing the 95% confidence intervals. The diamond symbol at the bottom reflects the combined effect size.

Acupuncture was found to moderately reduce the frequency and severity of dry mouth symptoms, as illustrated in Figure 5A (SMD: -0.618, 95% CI: -0.897 to -0.339; *I²* = 64.60%, *p* = 0.006). Subgroup analysis (Figure 5B) further indicated that acupuncture had a moderate impact on reducing symptom frequency and severity (SMD: -0.702, 95% CI: -1.040 to -0.364; *I²* = 68.84%, *p* = 0.004), while TENS demonstrated only a small effect (SMD: -0.360, 95% CI: -0.703 to -0.017; *I²* = 0%, *p* = 1). Additionally, acupuncture showed a moderate positive effect on quality of life, as shown in Figure 6A (SMD: -0.510, 95% CI: -0.780 to -0.239; *I²* = 67.45%, *p* = 0.005). Subgroup analysis (Figure 6B) confirmed that acupuncture provided a significant improvement in QoL (SMD: -0.593, 95% CI: -0.980 to -0.207; *I²* = 76.97%, *p* = 0.002), whereas TENS demonstrated only a minimal effect (SMD: -0.365, 95% CI: -0.645 to 0.086; *I²* = 0%, *p* = 0.389). These results highlight acupuncture's moderate efficacy in alleviating dry mouth symptoms and enhancing quality of life, while TENS exhibits a comparatively smaller impact.

### Publishing bias

**Figure 7:**
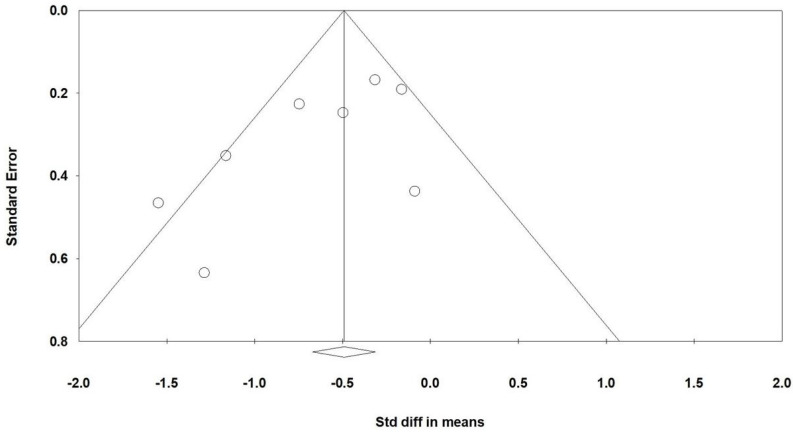
** Shows a funnel plot summarizing the results from the studies included in Figure 3A.** The lines illustrate the confidence intervals around the effect estimates, indicating the range where the true effect size is likely to fall. Circles represent individual studies included in the meta-analysis, with their size potentially reflecting the weight or sample size of each study; larger circles correspond to studies with greater weight or larger sample sizes. The diamond symbol denotes the overall effect estimate from the meta-analysis, with the center marking the pooled effect size and the width indicating the confidence interval for this estimate.

Funnel plots (Figure 7) illustrate the standardized mean differences for the effectiveness of acupuncture interventions in improving RSF among patients with radiation-induced xerostomia, as shown in Figure 3A. Egger regression analysis revealed minimal publication bias (p = 0.078).

## Discussion

This meta-analysis evaluated the efficacy of acupuncture in managing radiation-induced xerostomia, revealing moderate improvements in both resting and stimulated salivary flow rates, as well as reductions in the frequency and severity of dry mouth symptoms. Acupuncture also demonstrated significant enhancements in patients' quality of life. These findings align with previous studies that suggest acupuncture's potential to stimulate salivary gland function through autonomic nervous system activation and local vascular modulation.

Our results showed that acupuncture had a moderate effect on salivary flow rates and patient-reported outcomes, with consistent improvements across subgroup analyses. These effects are clinically meaningful, as increased salivary flow contributes to better oral hydration and reduced discomfort for patients undergoing radiotherapy. Furthermore, improvements in quality of life highlight the broader impact of acupuncture on patient well-being, beyond symptom management. Acupuncture's impact on RSF may be attributed to its ability to activate parasympathetic pathways 25-27, which are crucial for basal salivary secretion. By targeting specific acupoints associated with autonomic regulation, such as LI4 (Hegu), SP6 (Sanyinjiao), and CV24 (Chengjiang) 9, acupuncture may enhance the baseline activity of salivary glands. Moreover, the neurovascular effects of acupuncture, including increased local blood flow 28 to the parotid and submandibular glands 20, 29, likely contribute to improved glandular function and RSF. The improvement in SSF reflects acupuncture's ability to augment salivary production under stimulated conditions, such as chewing or gustatory responses 30. This could be mediated through the activation of central neural pathways that enhance the responsiveness of the salivary glands. Additionally, acupuncture-induced modulation of inflammatory cytokines, such as TNF-α and IL-6, may reduce glandular inflammation and fibrosis caused by radiation therapy, further improving stimulated salivary output 31, 32.

The significant improvements in RSF and SSF underscore acupuncture's utility as a comprehensive treatment for radiation-induced xerostomia. Resting salivary flow is essential for maintaining oral hydration and mucosal health 33, while stimulated salivary flow is crucial for functions like chewing, swallowing, and speaking 34. By addressing both aspects, acupuncture provides multifaceted relief for patients, enhancing their overall quality of life. Further studies should aim to explore the long-term sustainability of RSF and SSF improvements following acupuncture. Additionally, research into optimal treatment regimens, including the duration and frequency of sessions, could enhance the therapeutic potential of acupuncture for xerostomia management.

Subgroup analyses revealed that traditional acupuncture outperformed transcutaneous electrical nerve stimulation (TENS) in all primary and secondary outcomes. While TENS showed small effects on salivary flow and quality of life 35, the lack of significant improvements suggests that traditional acupuncture may engage more complex mechanisms, such as deeper neuromodulation or enhanced blood flow to salivary glands 25, 26. These findings indicate that traditional acupuncture should remain the preferred modality for managing radiation-induced xerostomia. The observed differences in efficacy may be attributed to the distinct mechanisms of action of acupuncture and TENS. Acupuncture involves needle insertion into specific acupoints, which may activate both the autonomic nervous system and local neurovascular pathways 36, leading to enhanced salivary gland function. It also stimulates deep structures, potentially eliciting a more profound systemic response 32. In contrast, TENS delivers electrical impulses through the skin, targeting superficial nerves without penetrating deep tissues 35. This difference could explain the comparatively limited effects of TENS, particularly in addressing complex symptoms such as dry mouth severity and quality of life. While TENS may still have a role, particularly in settings where acupuncture is unavailable or for patients who are averse to needles, its limited efficacy underscores the need for careful consideration of its application.

Moderate to high heterogeneity (I² ranging from 55% to 77%) was observed in several outcomes, likely reflecting differences in study protocols, acupuncture techniques, and patient characteristics. Additionally, Egger's regression analysis suggested potential publication bias (p = 0.078). The asymmetry in funnel plots indicates the possibility of unpublished studies with negative results. This bias could overestimate the reported effects, underscoring the need for future research to include unpublished trials or grey literature to minimize bias.

This meta-analysis has several limitations. First, the variability in acupuncture protocols, including point selection, session duration, and frequency, may contribute to heterogeneity and affect the generalizability of results. Second, the lack of blinding in most included trials raises concerns about detection bias, as outcome assessors were aware of treatment allocations. Third, the limited number of trials investigating TENS restricts the ability to draw definitive conclusions about its efficacy relative to acupuncture. The small effects observed for TENS could be influenced by the variability in electrical parameters, such as frequency and intensity, across studies. Additionally, the superficial stimulation provided by TENS may not be sufficient to trigger meaningful changes in salivary gland activity for patients with severe radiation-induced damage. Fourth, the inclusion of RCTs from countries such as Sweden, Brazil, South Korea, the United States, China, the UK, and Canada enhances this meta-analysis by offering a comprehensive, global perspective on the use of acupuncture for managing radiation-induced xerostomia. However, the variation in patient populations, healthcare systems, and methodological approaches introduces potential heterogeneity that must be carefully addressed. Conducting subgroup analyses, where possible, could provide valuable insights into whether acupuncture's effectiveness differs across regions or specific population characteristics.

## Conclusion

This meta-analysis provides robust evidence supporting the effectiveness of acupuncture in improving radiation-induced xerostomia. While heterogeneity and potential publication bias should be considered, the consistent benefits observed across multiple outcomes highlight acupuncture's value as a non-pharmacological intervention in this patient population.

## Supplementary Material

Supplementary figures and tables.

## Figures and Tables

**Table 1:** Characteristics of included studies

Author (year) / Country	Inclusioncriteria	Exclusion criteria	Sample size(% of male)/age	Studydesign	Placebo using	Intervention/ Duration	Main Results	Secondary Results
Blom (1996) / Sweden	1. Received radiotherapy for head and neck cancer (50-68 Gy in fractions of 2 Gy daily, 5 days a week).2. All patients reported having a subjectively dry mouth.	Not explicitly mentioned.	Acupuncture: 20 (70)/ Placebo: 18 (66)/Acupuncture: 61.5 Placebo: 64	RCT/ Single-blinded/ Placebo	Superficial acupuncture (intradermal needling 1 cm away from classical acupuncture points) without stimulating a needle reaction.	1. Two series of 12 acupuncture treatments, each lasting 20 minutes, performed twice a week for 6 weeks, followed by a 2-week pause between series.2. Total intervention period was approximately 14 weeks, followed by a 1-year observation period.	1. Improved salivary flow rates.2. After treatment, 68% of the experimental group increased salivary flow rates.3. Improvement persisted during the 1-year observation period.	Improvement was seen in other symptoms like decreased mucus secretion, improved taste, reduced pain in the tongue, diminished hoarseness, and better general health (including improved sleep and appetite).
Cho (2008)/ South Korea	1. With xerostomia who had received radiotherapy (minimum dose >38 Gy with at least 50% of the parotid glands exposed).2. Patients with head and neck cancers.	1. Distant metastases or inflammatory diseases requiring medical treatment.2. Eastern Cooperative Oncology Group (ECOG) performance status >2.	Acupuncture: 6 (83)Sham: 6 (83)/Acupuncture: 44 Sham: 44	RCT/ Single-blinded/ Placebo	Sham acupuncture at non-acupoints, with superficial needling (less than 0.5 cm depth).	Acupuncture was performed twice weekly for 6 weeks.	1. Acupuncture group showed significant improvement in RSF rates at 6 weeks compared to baseline (*p* < 0.05).2. No significant differences between the groups for SSF rates.3. Acupuncture group also significantly improved subjective xerostomia symptoms, increasing the questionnaire score (*p* < 0.05).	Acupuncture group improvements in xerostomia-related symptoms, such as dry mouth, eating, and speaking difficulties, as well as better sleep quality.
Braga (2011) / Brazil	1. Squamous cell carcinoma of the head and neck.2. Receiving primary or postoperative external beam radiotherapy (> 5000 cGy) involving >50% of the major salivary glands bilaterally.3. Concomitant chemotherapy allowed.	No mentioned.	Acupuncture: 12 (75)Sham: 12 (58.3)/Acupuncture: 64.5 Sham: 58	RCT/ Placebo	No acupuncture	Twice weekly for 16-20 sessions, following principles of traditional Chinese and Western medicine, focusing on local, distal, and auricular points/ 10 weeks.	1. Significantly higher salivary flow rates both for RSF and SSF (*p* < 0.001 for both).2. Lower scores on xerostomia-related symptoms measured by the Visual Analog Scale, with improvements in speaking, swallowing, and mouth dryness.	No significant adverse effects from acupuncture were reported, except for occasional somnolence and minor hematomas at the needle sites.Subjective improvements were also observed in oral and systemic symptoms, including reduced pain and dysgeusia, and improved sleep quality.
Meng (2012) / China and United States	1. Nasopharyngeal carcinoma undergoing radiation therapy.2. Anatomically intact parotid and submandibular glands.3. Zubrod performance status of 0, 1, or 2.	1. History of xerostomia planned IMRT, physical closure of salivary gland ducts, or contraindications for acupuncture.2. Taking medications or herbs affecting salivary function.	Acupuncture: 39 (76.9) Sham: 45 (64.4)/Acupuncture: 45.6 Sham: 48.9	RCT/ Placebo	Standard care	Acupuncture was administered at specific points (Ren 24, Lung 7, Kidney 6, Shenmen, Point Zero, Salivary Gland 20, and Larynx) 3 times per week during radiation therapy/ 7 weeks	1. Significantly lower xerostomia scores (based on the Xerostomia Questionnaire) from week 3 and continuing up to 6 months after radiotherapy (*p* < 0.0001).2. By 6 months, only 24.14% of the acupuncture group reported clinically significant xerostomia compared to 63.6% in the control group (*p* = 0.0018).	1. Better overall QoL and significantly higher SSF rates at 6 months compared to the control group (*p* < 0.003).2. There were no significant adverse events related to acupuncture, making it a safe and well-tolerated intervention.
Meng (2012) / China and United States	1. Aged 18 years or older diagnosed with nasopharyngeal carcinoma.2. Intensity-modulated radiation therapy with intact parotid and submandibular glands.3. Zubrod performance status of 0, 1, or 2.	1. History of xerostomia, physical closure of salivary gland ducts, known bleeding disorders, or spinal cord injury.2. Taking medications or herbs affecting salivary function or planning to take such substances during the study.	Acupuncture: 10 (90)Sham: 11 (100)/Acupuncture: 45.8 Sham: 47.2	RCT/ Placebo	Sham acupuncture with a validated non-penetrating needle device placed at non-active points.	Real acupuncture involved 18 sessions, 3 times weekly, using specific body and ear points/ 6 weeks	1. Significantly lower Xerostomia Questionnaire scores starting from week 3 compared to the sham group (*p* < 0.006), with sustained improvements through week 11.2. At week 11, only 12.5% of the acupuncture group had clinically significant xerostomia compared to 75% in the sham group (*p* = 0.02).	1. No significant differences in RSF and SSF rates between the groups.2. Improved symptom burden scores from week 3 to week 6, with significant differences in head and neck symptom scores (*p* < 0.0001).
Simcock (2013) / UK	1. Received radical radiotherapy for head and neck cancer, with at least one parotid gland in the radiation field.2. Rrequired to be recurrence-free for at least 18 months post-treatment.	1. History of heart valve disease, bleeding disorders, frequent infections, or needle phobias.2. Reconstructive prostheses or surgical scars at acupuncture points.	Acupuncture: 70 (77)Sham: 74 (73)/Acupuncture: 58.6 Sham: 60.3	RCT/ crossover trial/ Placebo/ Randomized receive either oral care followed acupuncture or acupuncture followed by oral care.	Oral care education.	Acupuncture: 8 weekly group sessions, each lasting 20 minutes, using standardized acupuncture points (ear and body points including LI2 and LI20).Oral care education: 2 group sessions (one at baseline and one a month later), focused on the etiology of xerostomia, dietary advice, and oral hygiene/ 8 weeks	1. Significant reductions in symptoms such as severe dry mouth (*p* = 0.031), sticky saliva (*p* = 0.048), the need to sip fluids to swallow food (*p* = 0.011), and waking up at night to drink (*p* = 0.013).2. No significant changes in either stimulated or unstimulated saliva production were observed over time.	1. Female participants were more likely to report severe dry mouth (*p* = 0.03), and subjective symptoms improved with acupuncture more than oral care.2. Despite improvements in subjective symptoms, no correlation was found between the improvements and actual salivary flow rates.3. QoL scores did not significantly change over the course of the trial, and no significant adverse events related to acupuncture were reported.
Wong (2015) / United States and Canada	1. Aged 18 or older, who completed radiation therapy 3 months to 2 years prior, without evidence of recurrence.2. Grade 1 or higher xerostomia (CTCAE v3.0), and with a residual basal salivary production > 0.1 ml/min.	1. Contraindications to pilocarpine or ALTENS. 2. Previously treated with pilocarpine or cevimeline had to discontinue these medications 2 weeks prior to randomization.	ALTENS: 73 (84.9), Sham: 73 (86.3)/ALTENS: 58Sham: 59	RCT/ Placebo	Pilocarpine.	ALTENS: Delivered with TENS units to acupuncture points for 24 sessions (twice weekly) over 12 weeks.Pilocarpine: 5 mg, orally, three times daily for 12 weeks.	1. At 9 months, the mean change in the Xerostomia QoL Scale (*p* = 0.45).2. At 15 months, the response rate (>20% improvement in xerostomia burden).3. Both groups showed no significant difference in whole salivary production.	1. ALTENS was associated with fewer adverse events (20.8%).2. ALTENS group demonstrated greater treatment compliance (93%).
Wyatt (2016) / United States and Canada	1. Aged 18 or older.2. Completion of radiation therapy (IMRT or standard conformal) with or without chemotherapy 3 months to 2 years before study entry.3. No evidence of head/neck disease recurrence and disease-free from other invasive malignancies for at least 3 years prior.4. Reporting grade 1 or higher xerostomia (CTCAE v3.0) with a residual basal salivary production > 0.1 ml/min.5. Zubrod performance status of 0 to 2.	Unstable cardiac disease, in-situ pacemakers, respiratory illnesses requiring hospitalization, bacterial/fungal infections requiring IV treatments, or pregnancy.	ALTENS: 69 (85.5)Sham: 68 (86.8)/ALTENS: 58Sham: 58.5	RCT/ Placebo	Pilocarpine.	ALTENS: Delivered using a Codetron™ unit to bilateral acupuncture points (SP6, ST36, LI4, CV24), administered 2 times per week for 12 weeks.Pilocarpine (PC): 5 mg orally, three times daily for 12 weeks.	Both groups improvement in radiation-induced xerostomia over time.	1. ALTENS was associated with fewer side effects. 2. No significant improvements in whole salivary production were observed.
Garcia (2019) / United States and China	1. Oropharyngeal or nasopharyngeal carcinoma.2. Undergoing radiation therapy, with or without chemotherapy, and a mean dose of >24 Gy to at least one parotid gland.	1. Prior acupuncture experience or missing intact parotid glands.2. Participating in another randomized clinical trial or unable to accommodate the study schedule.	Acupuncture: 112 (77.6) Sham: 115 (77.6) Standard care: 112 (77.6)/Acupuncture: 51.3 Sham: 51.3 Standard care: 51.3	RCT/ three arm clinical trial/ Placebo	Sham acupuncture with a validated non-penetrating telescopic needle placed at non-active points.	True acupuncture and sham acupuncture were administered 3 times per week/ 7 weeks	1. One year after radiation therapy, the adjusted xerostomia score was significantly lower in the acupuncture group.2. Significant xerostomia was lowest in the acupuncture group.	Improved quality of life and reduced xerostomia symptoms.
Paim (2019) / Brazil	1. Head and neck cancer who completed radiotherapy at least 90 days prior.2. No tumor lesions in salivary glands and skin integrity in the area of electrode placement.	1. Stimulated salivary flow above 1.0 mL/min, severe dysphagia, or undergoing other hyposalivation treatments.2. Pacemakers or any other contraindications for TENS.	TENS group: 37 (94) Sham: 31 (100)/ TENS group: 57.5 Sham: 59.9	RCT/ Placebo	Sham TENS	TENS administered for 8 sessions over 4 weeks (twice weekly), with each session lasting 20 minutes/ 4 weeks	1. Significant increase in SSF from the third session onward, with continuous improvement up to 6 months (*p* < 0.0001).2. SSF and self-perception of salivary flow (SPSF) significantly improved, with a lasting impact through the 6-month follow-up.	1. QoL significantly improved, with better scores in speech, deglutition, mastication, and saliva satisfaction.2. No significant adverse events were associated with TENS therapy, indicating its safety and tolerance.
Cohen (2024) / United States	1. Aged 18 years or older with head and neck cancer who received first-line bilateral external-beam radiotherapy with curative intent >12 months prior to enrollment.2. Anatomically intact parotid glands and at least one submandibular gland.3. Grade 2 or 3 xerostomia per RTOG scale, related to radiotherapy.	1. History of xerostomia, Sjögren syndrome, or other illness known to affect salivation prior to radiotherapy.Active systemic or skin infections at or near acupuncture sites.2. Currently receiving other xerostomia treatments or investigational drugs, or chemotherapy during the study period.	Acupuncture: 86 (79) Sham: 86 (77.9) Standard care: 86 (76.7)/Acupuncture: 65 Sham: 64.4 Standard care: 65.6	RCT/ Single-blinded/ three-arm placebo-controlled trial.	Sham acupuncture with non-penetrating telescoping needles placed at non-active points.	True acupuncture: 14 body and ear points (3 points per ear and various body points). Administered twice weekly for 4 weeks.Sham acupuncture: Non-penetrating needles at inactive points, following the same schedule as the true acupuncture group.	1. Significant improvements in xerostomia scores (*p* = 0.003).2. Improvements in quality of life (*p* = 0.002).	No significant differences in adverse events across groups.

ALTENS: acupuncture-like transcutaneous electrical nerve stimulation; RCT: randomized controlled trial; RTOG: radiation therapy oral mucositis grading; OMAS: Oral Mucositis Assessing Scale; TENS: transcutaneous electrical nerve stimulation. SSF: stimulated salivary flow; QoL: quality of life; RSF: resting salivary flow; IMRT: intensity-modulated radiation therapy.
